# School Plays a Crucial Role in Children’s Healthy Nutrition and Lifestyle

**DOI:** 10.3390/nu17132169

**Published:** 2025-06-30

**Authors:** Josep A. Tur, Marcela González-Gross

**Affiliations:** 1 CIBER Fisiopatología de la Obesidad y Nutrición (CIBEROBN), Instituto de Salud Carlos III (ISCIII), 28029 Madrid, Spain; 2 Research Group on Community Nutrition and Oxidative Stress, University of the Balearic Islands-IUNICS, 07122 Palma de Mallorca, Spain; 3 Health Research Institute of the Balearic Islands (IdISBa), 07120 Palma de Mallorca, Spain; 4 Department of Health and Human Performance, Universidad Politecnica de Madrid, 28003 Madrid, Spain

A large part of the lives of children and adolescents takes place at school [1]. Educational centers can exert a great influence on the lifestyle of children and adolescents. Therefore, no one doubts that schools are crucial in promoting the present and future health of children and adolescents, mainly by implementing or promoting healthy eating and physical activity habits in their daily lives. [2,3].

This Special Issue shows the latest results of studies oriented in this direction, including original research, as well as special reports on recommendations and experiences to apply locally in schools, but also addressed to the managers and authorities responsible for public health nutrition.

An assessment of the association between school lunch and body size in Japanese junior high school students opened this Special Issue (Contribution 1). Through a cross-sectional study, it was stated that just 65.6% of junior high school students lunched at school, despite no significant associations being found for normal weight and overweight/obesity or underweight outcomes, showing the null effect of the present Japanese school lunch program.

A second paper assessed the dietary and nutritional quality of the menus served in Spanish Defense Ministry preschool education centers (1–3-year-old children) to ascertain their compliance with dietary and nutritional recommendations (Contribution 2). The assessed menus were correct in energy values, but the energy profile was higher than recommended in protein and fat while being lower in carbohydrates, whereas the lipid profile was adequate, but high in cholesterol, simple sugars, fiber, vitamin K, selenium, potassium, and sodium, and deficient in omega-3 fatty acids, vitamin D, iodine, and zinc.

The PESCA program (Spanish acronym that means scholar program of cardiovascular health) was a multicomponent school-based intervention launched in 2018 to tackle the health problem of aiming to reduce overweight rates in youth (Contribution 3). It showed that this program positively impacted physical activity/sport practice prevalence and provided protection against overweight and related variables during the pre-COVID period.

Despite the fact that the public procurement of food is crucial for ensuring proper nutrition and the provision of high-quality products in public institutions like schools and kindergartens, the cheapest and often lowest-quality products are usually selected. To ascertain the quality of foods in Polish kindergartens, the quantities ordered and the quality characteristics were assessed (Contribution 4). Generally, the sensorial characteristics (consistency and color) were specified, underscoring minimum standards upon observation of composition and sustainability.

The Netherlands Healthy School Program stimulates a healthier dietary intake for students through schools to improve the adherence of students to dietary guidelines (Contribution 5). The best results among primary schools were obtained when the program was implemented in high-socioeconomic-status schools through a combination of high implementation and parental and student support. In secondary schools, the program resulted in a healthier dietary intake only if the environment was not a hindrance and low socioeconomic status was not an issue.

Observing the recommendations for salt intake is crucial for a healthy life (Contribution 6). It was reported that as children get older, the impact of parents and familial socio-environmental factors begins to wane, and other factors start to have an influence, specifically school education on healthy lifestyle habits and the health behavior of peers.

A healthy lifestyle is crucial for safeguarding the well-being and quality of life perception, appropriate growth, and development of children and adolescents (Contribution 7). Accordingly, associations between perceived quality of life and a healthy lifestyle and related outcomes in Spanish children and adolescents were assessed. Participants with a higher health-related quality of life were those with a higher adherence to the Mediterranean Diet, a higher intake of the recommended daily amount of fruit and vegetables, better recommendations for screen time and sleep, a higher healthy weight status, and higher physical fitness.

The Zeliakide Project (Contribution 8) is a nutrition education program based on inquiry-based learning, aimed at increasing the general population’s knowledge about healthy diets, celiac disease, and a lifelong gluten-free diet and improving the social inclusion and quality of life of people with celiac disease. It has been demonstrated to be a valid and useful tool for achieving changes in diet at the school level and will help to promote the social inclusion of people with celiac disease.

All of these studies show that school is essential for acquiring healthy diet and activity habits. Accordingly, both the activity of managers of educational centers, as well as the characteristics and nutritional offerings of school canteens, must be regulated [4,5] and, finally, why not honestly questioned and evaluated? Our descendants, children, and adolescents will appreciate these recommendations for their health, which will result in the public health of future members of society.
